# In search of low-frequency and rare variants affecting complex traits

**DOI:** 10.1093/hmg/ddt376

**Published:** 2013-08-06

**Authors:** Kalliope Panoutsopoulou, Ioanna Tachmazidou, Eleftheria Zeggini

**Affiliations:** Wellcome Trust Sanger Institute, Hinxton, UK

## Abstract

The allelic architecture of complex traits is likely to be underpinned by a combination of multiple common frequency and rare variants. Targeted genotyping arrays and next-generation sequencing technologies at the whole-genome sequencing (WGS) and whole-exome scales (WES) are increasingly employed to access sequence variation across the full minor allele frequency (MAF) spectrum. Different study design strategies that make use of diverse technologies, imputation and sample selection approaches are an active target of development and evaluation efforts. Initial insights into the contribution of rare variants in common diseases and medically relevant quantitative traits point to low-frequency and rare alleles acting either independently or in aggregate and in several cases alongside common variants. Studies conducted in population isolates have been successful in detecting rare variant associations with complex phenotypes. Statistical methodologies that enable the joint analysis of rare variants across regions of the genome continue to evolve with current efforts focusing on incorporating information such as functional annotation, and on the meta-analysis of these burden tests. In addition, population stratification, defining genome-wide statistical significance thresholds and the design of appropriate replication experiments constitute important considerations for the powerful analysis and interpretation of rare variant association studies. Progress in addressing these emerging challenges and the accrual of sufficiently large data sets are poised to help the field of complex trait genetics enter a promising era of discovery.

The genetic architecture of complex traits has not been fully elucidated yet. Following the advent of genome-wide association studies (GWASs) and large-scale consortial meta-analyses of GWASs, several thousands of variants have been robustly associated with complex phenotypes of medical relevance (http://www.genome.gov/gwastudies), giving valuable insights into underlying biological processes. GWASs are designed to provide a survey of common variation [minor allele frequency (MAF) > 0.05], therefore examining only a portion of the genomic landscape of complex traits. Low-frequency (MAF 0.01–0.05) and rare (MAF < 0.01) variation has thus far been more challenging to access. Early studies on data from deep sequencing of small numbers of loci and more recently larger-scale studies (e.g. 1000 Genomes Project) demonstrate that rare variants constitute the majority of polymorphic sites in human populations (1–3).

## ACCESSING RARE VARIANTS

**Figure 1.**
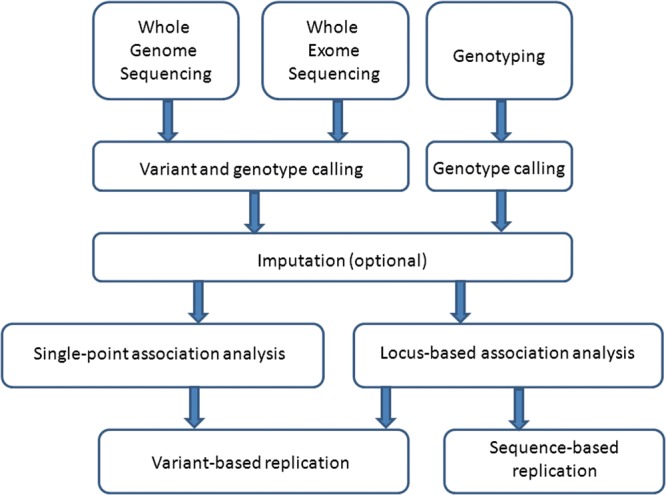
An overview of steps taken in the search for low-frequency and rare variants affecting complex traits.

Current approaches to investigate the effect of rare variants in complex traits involve direct genotyping—for example, through targeted arrays like the exome chip (http://genome.sph.umich.edu/wiki/Exome_Chip_Design), metabochip (4) or immunochip (5), using the GWAS as a scaffold to impute low-frequency variants based on a sequenced reference panel (e.g. 1000 Genomes Project) (3), or resequencing of specific regions and increasingly the whole exome (WES) or the whole genome sequencing (WGS) (schematic overview in Fig. 1). The most commonly used WGS platforms generate millions of short sequence reads that are then aligned to a reference genome through read mapping. Variant calling algorithms are subsequently employed to identify candidate sites at which one or more samples differ from the reference sequence and to call genotypes across samples. Currently, studies tend to focus on single nucleotide variants, as accurate calling of copy number variation is less straightforward. High-depth WGS is currently the preferred approach to exhaustively study variation across the full allelic spectrum genome-wide but for complex trait studies, where a large number of individuals need to be sampled costs remain prohibitively expensive. For mapping complex trait variants, study designs that increase the number of sequenced samples by decreasing sequencing depth are more powerful and cost-effective than sequencing fewer individuals at high depth (4,6–8), but detection and calling accuracy at rare variant sites can be compromised. WES interrogates only the coding regions of genes. The lower cost of WES compared with WGS means that higher depths are feasible, leading to higher accuracy in rare variant calls.

## EXAMPLES OF RARE VARIANTS CONTRIBUTING TO COMPLEX TRAITS

There is growing evidence that rare variants can play a role in complex disease aetiology. One of the earliest examples came from the field of breast and ovarian cancers with the discovery of multiple rare mutations in the *BRAC1* and *BRAC2* genes (reviewed in 9–11). A more recent example is provided by a study which identified four rare variants acting independently on type 1 diabetes (T1D) risk through targeted resequencing of *IFIH1*, a gene located in a region previously associated with T1D by GWAS (12). A further report of rare variants exerting individual effects showed that five distinct rare variants in *NOD2* are associated with the risk of Crohn's disease and appear to act independently from each other and from the previously implicated low-frequency causal variants (13–15). In a different allelic architecture paradigm, sequencing the exons of the GWAS-implicated type 2 diabetes (T2D) gene *MTNR1B* identified several rare variants impairing melatonin receptor 1B function which collectively contribute to T2D risk (16). Several examples of rare and low-frequency variants with individually large effect sizes have also been reported. For example, a rare missense variant in *MYH6* was found to be associated with high (∼12-fold) risk of sick sinus syndrome (17) in a pioneering study from Iceland, which combined whole-genome sequencing, genome-wide genotyping and imputation-based approaches. Notably, over the past few years, WGS of affected trios has led to the identification of several *de novo* mutations implicated in the aetiology of autism (18–21), schizophrenia (22,23) and intellectual disability (24).

Perhaps, the most abundant examples of rare variants acting collectively have emerged from the study of medically relevant quantitative traits and in particular circulating lipid levels. Screening for variants in genes implicated in Mendelian forms of low high-density lipoprotein cholesterol (HDL-C) levels revealed an aggregation of rare alleles in individuals with low HDL-C compared with those with high HDL-C (25). Resequencing of *ANGPTL4* uncovered both rare and common variants that reduce triglycerides and increase HDL (26). Recently, a region near *PARM1* was implicated in HDL-C level variation through a sliding-window burden testing approach performed on variants with MAF < 0.01 in one of the first WGS-based complex trait studies to date (27). An aggregation of rare alleles has also been associated with low-density lipoprotein cholesterol (LDL-C) (28) and with blood pressure reduction and protection against hypertension (29). The first reported application of exome array genotyping identified five independently acting, low-frequency variants associated with fasting proinsulin concentrations (30).

## POPULATION ISOLATES

The study of rare variation can be empowered by focusing on population isolates (31,32). Isolated populations are characterized by increased phenotypic, genetic and environmental homogeneity. In these populations, rare variants may have drifted up in frequency and linkage disequilibrium (LD) tends to be extended. Founder populations carry a subset of the genetic variation present in the original population from which they have diverged. The effect of random genetic drift on increasing allele frequencies is higher for rare compared with common variants. Population bottlenecks can also affect the genetic architecture of isolates by reducing the population size and hence heterogeneity, increasing endogamy levels and subsequently increasing levels of homozygosity and LD. Population isolates tend to demonstrate geographical and/or cultural isolation frequently commensurate with a homogeneous set of environmental exposures, e.g. diet and lifestyle.

For example, the Iceland-based deCODE study has been successful in identifying rare variants contributing to complex traits by leveraging these characteristics of population isolates in conjunction with extended genealogical information for a variety of complex traits (including prostate cancer, Alzheimer's disease, gout and serum uric acid levels) (33–36). Association of a rare functional variant (R19X) in the *APOC3* gene with HDL-C and triglycerides levels was first detected in the Amish founder population and later confirmed in a Greek population isolate from Crete. R19X appears to have drifted up in frequency independently in the two population isolates (37,38).

## RARE VARIANT REFERENCE PANELS

Large-scale collaborative efforts aiming to help understand the full spectrum of sequence variation serve as valuable resources for the scientific community. The 1000 Genomes Project (3) has helped enhance our understanding of low-frequency and rare variants by providing a catalogue of common and uncommon variation through WGS and exon sequencing across several global populations. Importantly, it has also enabled the first-line use of imputation approaches (39) to infer genotypes at untyped low-frequency variants in large-scale GWAS meta-analysis efforts. This approach has started contributing to the identification of novel disease-associated variants (e.g. 40). The National Heart Lung and Blood Institute Exome Sequencing Project (NHLBI-ESP) (https://esp.gs.washington.edu) has provided insights into rare coding variants through WES of 6500 samples in phenotyped sets from the USA. The UK10K Project (www.uk10k.org) has undertaken high-depth WES of 6000 and low-depth WGS of 4000 well-phenotyped individuals primarily from the UK. The imputation of low-frequency and rare variants can be challenging compared with common alleles, and the availability of very large-scale reference panels can improve imputation performance. It is envisaged that large-scale WGS efforts will join forces to generate an overarching reference panel to enable efficient and widespread use of the generated data.

## RARE VARIANT ASSOCIATION ANALYSIS

Statistical genetics considerations of rare variant association analysis have been the focus of intensive method development over the last few years. The single-point analysis of rare variants is under-powered, because not enough copies of the rare variant allele are observed in sample sizes typically available to date. Instead of examining the association of each rare variant in isolation, multivariate methods that combine information across multiple variant sites within a gene or other functional genomic region are a viable alternative strategy (Fig. 1). A plethora of such locus-specific statistical approaches have been developed and fall broadly into a few categories: collapsing methods based on summary statistics (Cohort Allelic Sum Test (41); Combined Multivariate and Collapsing Test (42); Weighted Sum Test (43); Variable-Threshold Approach (44)); methods based on similarities among individual sequences (Kernel Based Association Test (45); Sequence Kernel Association Test (46)); and regression models that use collapsed sets of variants and other factors as predictors (collapsing test using proportion of rare variants (47); Adaptive Sum Test (48); LASSO and Ridge Regression (49)) (50).

Collapsing methods aggregate information across multiple variants within a region of interest into a single quantity, which is then used to test for trait association with an accumulation of rare minor alleles. Collapsing methods vary in the way they collapse the variants and in the chosen statistical test (42,47), for example options include using a regression approach that models the phenotype as a function of the proportion of rare variants at which an individual carries a minor allele, or as a function of the presence or absence of a minor allele at any rare variant within an individual. Collapsing approaches assume that all collapsed variants are associated with the disease, and that they can be either deleterious or protective. Alternative approaches that model similarities among individual sequences using various kernel functions, such as KBAT (45) and SKAT (46), are multivariate tests that combine single-variant test statistics. They make no assumptions about the probability or direction of effect of each rare variant, and are therefore more flexible, given that the allelic architecture of complex traits is unknown. A unified approach between collapsing methods and SKAT (SKAT-O (51)) adapts to the data to give more weight to the test that makes the most realistic assumptions for the specific region and trait of interest.

Genotype uncertainty metrics for imputed genetic variants or for sequencing-derived variants could be incorporated as weights in different statistical tests, instead of filtering out variants with low imputation or quality scores, to increase association power as shown by (52). Moreover, variants in rare association tests can be down or up weighted according to their probability of being functional. Such weights can be based on the MAF under the assumption that rarer variants are more likely to be deleterious according to the natural selection theory. Alternatively, weights can be based on functional annotation predictions. Coding variants that are predicted to have severe functional consequences may be hypothesized to confer larger phenotypic effects, have higher translational potential and may be more amenable to designing downstream functional experiments. Functional annotation of non-coding variation is more challenging and an active area of current research.

An open question for rare variant analysis in WGS studies is how to define the region of interest. In WES studies, such a decision is more straightforward, as a gene unit is an intuitive option. In WGS studies, a potential approach is to divide the genome into windows of certain physical size. However, it is not clear what the size of the windows should be and whether they should be overlapping or by how much. Genome-wide significance levels for the GWAS era were estimated to be at *P* = 5 × 10^−8^ based on the number of independent common-frequency variants across the genome calculated based on the European population data from the HapMap Project (53). This threshold has served the scientific community well, representing a standard to be attained before declaring significance. The analysis of rare variants across the genome requires a more stringent significance threshold that takes into account single-point common and rare variant tests as well as burden tests. This threshold is likely to vary depending on the study design parameters like sample size and sequencing depth, and is expected to be lower for African-descent populations.

## META-ANALYSIS OF RARE VARIANTS

Meta-analysis of common variants in GWASs is a common strategy of combining studies examining the same trait to increase power to obtain statistical evidence of association. Traditional meta-analysis techniques, such as Fisher's (54) and Stouffer's (55) tests, that use region-level *P*-values from the different burden tests are not necessarily powerful in combining data across independent studies for rare variant association testing, as they do not capture all of the available information (56). Ideally, meta-analytical approaches for next-generation sequencing studies should result in little or no power loss when compared with a joint analysis approach, in the same way as meta-analysis of single tests for common variants (57).

Lumley *et al.* (58) and Lee *et al.* (59) have independently developed meta-analytical techniques for SKAT (46) and SKAT-O (51), respectively. The latter approach can assume both homogeneous and heterogeneous genetic effects across studies, corresponding to a fixed- and random-effects meta-analysis model, respectively. Both approaches are similar in spirit and are based on study-specific summary statistics rather than individual-level data. They combine single-variant score statistics first across studies and then within a region. They also require between-variant covariance-type relationship statistics (such as LD structure) for each region, as well as MAF of variants.

Liu *et al* (60) and Tang and Lin (61) suggest approaches that encompass a number of popular gene-level association tests such as collapsing tests (42,47), variable threshold (44) and SKAT (46). Their methods also combine single-variant score statistics across studies. A unique feature of the Liu *et al* (2013) approach is that apart from calculating asymptotic *P*-values, it also evaluates significance in an empirical and numerically stable way via an adaptive Monte-Carlo simulation scheme. Another unique feature of Liu et al.'s approach is its ability to conduct conditional meta-analysis of gene-level tests. Lumley *et al.*, Lee *et al.* and Liu *et al.* (2013) show in simulation studies that their proposed approaches are as efficient as an analysis that pools individual-level data together. The evaluation of different meta-analysis approaches of rare variant tests is an active field of study.

## POPULATION STRATIFICATION AT RARE VARIANTS

Population stratification at rare variants is an important consideration for next-generation association studies. Rare variants show increased population specificity (3). Based on the theoretical examination, rare variants can show a stronger pattern of population stratification than common variants, particularly in the presence of sharp spatial distributions for non-genetic risk of disease (62). Existing methods to correct for population stratification at common variants such as principal component analysis and genomic control have not been shown to effectively control stratification at rare variants with implications for both single-point and locus-based approaches (62–64). In empirical data from the UK population, rare variants were found to display different stratification patterns to common variants (65). These findings underscore the need for carefully matching samples, for example cases and controls, between geographical regions and highlight the need for replication in independent datasets.

## REPLICATION OF RARE VARIANT SIGNALS

Strategies for replication of associations discovered in low-frequency and rare variants depend on the allelic architecture of the associated locus. For example, if a single low-frequency or rare variant is driving the signal, replication can be sought by genotyping the implicated variant in an independent sample set (e.g. 17). For associations uncovered via locus-based approaches, two replication strategies have been proposed: variant-based replication, where only variants found in the discovery phase are followed-up by, e.g. genotyping, and sequence-based replication, where the whole region is re-sequenced in the replication sample set and novel variants can be included in the test (Fig. 1). Under several simulation scenarios, it has been demonstrated that there are small gains in power when adopting the sequence-based replication design and more so if the discovery sample set is small. At medium- to large-scale studies and when discovery and replication sample sets are drawn from the same population, genotyping can offer a viable alternative solution (66).

The emerging generation of studies in search of low-frequency and rare variants affecting complex traits will require robust strategies to ensure high power in the context of an appropriate statistical framework. It is anticipated that sequence-based meta-analysis across diverse populations, including populations of African descent, will empower novel locus discovery, and that accruing the necessary sample sizes is likely to be a key determinant of success. Initial insights into the contribution of rare variation indicate a firm role in complex trait aetiology and suggest a combination of potential allelic architectures underpinning biological phenotypes. Their powerful detection will require tailored study design and analysis approaches.

## FUNDING

K.P., I.T. and E.Z. are funded by the Wellcome Trust (098051). K.P. is funded by Arthritis Research UK (19542). Funding to pay the Open Access publication charges for this article was provided by The Wellcome Trust (098051).
